# Household Hints and Recipes

**Published:** 1885-07

**Authors:** 


### Household Hints & Recipes.

GLACIAL ACETIC ACID dissolves almost all protein bodies, especially albumen and fibrin, but not casein. It, of course, destroys warts and corns.

FURNITURE POLISH.—

| | |
|---|---|
| Beeswax, finely divided | 2 ounces |
| Turpentine, | ½ pint. |
| Alkanet root, | ¼ ounce. |

Digest the alkanet root in th oil of turpentine. —*Druggist Circular.*

A HARMLESS SOLDERING MIXTURE.—A correspondent of thé *Sanitary Engineer* draws attention to the fact that a patent has recently been issued for a harmless liquid to be used in place of chloride of zinc solution for moistening tin plate to be soldered, particularly in the case of tin cans containing articles of food. The inventor is C. N. Waite, of Littleton, Mass. Its composition is equal parts by weight of *lactic acid* and *glycerin:*

TO RENDER FABRIC UNINFLAMMABLE.—The following is given as a cheap mode of rendering fabrics uninflammable : Four parts of borax and three parts sulphate of magnesia are shaken up together just before being required. The mixture is then dissolved in from 20 to 30 parts of warm water. Into the resulting solution the articles to be protected from fire are immersed, and when they are thoroughly soaked they are wrung out and dried, preferably in the open air.—*N. Y. Times.*

CHEAP AND GOOD COLOGNE.—*H. J. M. (Philadelphia, Pa.)* wishes a formula for a cheap and good cologne. Below we give a formula from "Perfumery and Kindred Arts" :

EAU DE COLOGNE.

| | |
|---|---|
| Oil of bergamot, lemon, orange and rosemary, of each 1 ounce | |
| Oil of cloves | 1 drachm |
| Tinct. of orris | 4 ounces. |
| Alcohol, | 1 gallon. |
| Water | 3 pints. |

This would probably do for a "cheap" cologne. Opinions differ so much in regard to perfumes that we would advise the writer to purchase some good work on this subject, like the one mentioned above, by Cristiani.—*Ex.*

HAIR AND WHISKER WAX.—Rosin 3 Venice turpentine 4 White Wax 12.

Melt, and perfume with volatile oils. —*Pharm. Zeitsch f Russi.* Nov., 1884, No. 48.

TO PRESERVE EGGS.—According to *Mittheilungen Landwirthschaft*, vaselin is a good preservative for eggs. The eggs should be thoroughly washed and rubbed with vaselin previously melted with three-tenths per cent. of salicylic acid.

The operation should be performed twice, the latter one month after the former. On boiling, the skin of vaselin easily separates from the eggs. Eggs thus treated are said to keep perfectly fresh for a year.

A PAINT FOR BLACKBOARDS which has been used in schools and elsewhere, and is found very satisfactory, is composed of 4 ounces of common glue, 3 ounces flour of emery, and sufficient lampblack to give the preparation an inky color. The glue is first dissolved in 1½ pints of water, the lampblack and emery are then put in, and the mixture stirred until all lumps have disappeared. The board should be given two or three coats, the paint being applied with a woollen rag smoothly rolled.

BLEACHING BONES.—By experiments made at the Bavarian Museum, a very simple and effective method of bleaching bones, to give them the appearance of ivory, has been discovered. After digesting the bones with ether or benzin to recover the fat, they are thoroughly dried, and immersed in a solution of phosphoric acid in water, containing one per cent. of phosphoric anhydride. After a few hours they are removed from the solution, washed in water, and dried, when they will appear as indicated above.—*Pop. Sci. News.*

MANUFACTURE OF ETCHING INK.—According to Muller, a liquid for etching on glass has recently been introdnced into commerce, and can be used with an ordinary pen. It consists of hydrofluoric acid, ammonium fluoride and oxalic acid, and is thickened with barium sulphate. A better ink is obtained as follows : equal parts of the double hydrogen ammonium fluoride and dried precipitated barium sulphate are ground together in a porcelain mortar. The mixture is then treated, in a platinum, lead, or gutta-percha dish, with fuming hydrofluoric acid, until the latter ceases to react.—*Dingl. Polyt.*

CHILBLAIN OINTMENT.—
Camphor, 1.
Balsam Peru, 1.
Simple cerate, 12.
—*Pharm. Zeitsch. f. Russl., Nov.*, 1884. *No.* 48.

TO CLEAN HEKTOGRAPH PADS.—Moisten the pad with a ten per cent. solution of hydrochloric acid [Acid Hydrochlor. Dil. U. S. P.—ED. D. C.] and rinse with pure water. This process will remove the ink and not injure the pad.—*National Druggist.*

LEMONADE EXTRACT.—
Citric acid. 8 parts.
Tinct. lemon, 1 part.
White sugar, 175 parts.
Water, 145 parts.
—*Pharm. Zeitsch. f. Russl-, Nov.* 1884, *No.* 48.

PERFUMED OIL FOR POMADES, HAIR OIL, ETC.—
Oil cloves, 3 parts.
Oil lemon, 12 parts.
Oil bergamot, 18 parts.
Oil thyme, 1 part.
The above 34 parts of essential oils to be mixed with finest olive oil 1,730 parts.—*Pharm. Zeitsch. f. Russl., Nov.*, 1884, *No.* 48.

CLEANING MORTARS.—Slodki, of Nancy, recommends the following method for cleaning mortars which have been used in making preparations of iodoform. After having washed the mortar, or if greasy, having cleaned it with saw-dust, a little alcohol is poured in, ignited, and stirred about with the pestle. When all the alcohol is consumed, the mortar and pestle are washed with water, and all trace of iodoform will then have disappeared.—*L' Union Pharm.*

TO CLEAN PLASTER-OF-PARIS BUSTS.—To clean casts of gypsum (plaster-of-paris), bas-reliefs, etc., which have stood for a time and have become dirty and dusty, several methods are employed, all of which, however, tend to injure the sharp corners of the cast, and occasion loss of time. The writer was required to do much of this work, and he hit on the following excellent plan: Boil a quantity of starch into a thick paste, and with a soft brush spread it on the article to be cleaned. Then expose it to an airy place, and, when dry, the paste will peel off, together with all the dirt and dust, and the cast will appear as white as when new.

DAMMAR VARNISH.—Dissolve 1 part of finely powdered and well dried dammar resin in 2 parts oil of turpentine.—*Pharm. Zeitsch. f. Russl., Nov.*, 1884, *No.* 48.

PERFUME FOR HAIR OILS AND POMADES.—
Oil Cinnamon, 3.
Oil cloves, 4.
Oil bergamot, 8.
Oil lemon, 15.
—*Pharm. Zeitsch. f. Russl., Nov.*, 1884, *No.* 48.

CLEANING-RAGS.—A French druggist transacted quite a lucrative trade with the sale of cleaning-rags, excellent for polishing metalic surfaces, which he prepared as follows: Dip flannel rags into a solution of 20 parts of dextrin and 30 parts oxalic acid in 20 parts logwood decoction, wring them gently, and sift over them a mixture of finely pulverized tripoli and pumice-stone. The moist rags are piled upon each other, placing a layer of the powder between each two. They are then pressed, taken apart, and dried.

LIQUID SOLDER FOR TIN WARE.—*C. E. D.* (*Ohio*).—The nearest approach to the desired information is the following;

SOLDERING WITHOUT HEAT.

| | |
|---|---|
| Flouric acid | ¼ ounce. |
| Brass filings | 2 ounces. |
| Steal filings | 1 ounce. |

Put the filings in the acid, and apply the solution to the parts to be soldered, which dress together. (Keep the acid in an earthern vessel.)

The clipping below, from the *American Druggist*, may be of value.

RUBBER STAMPS.—To make these, the name or whatever is required is first set up in ordinary type and in the style required. A rim about ½ inch high is placed around the form, and dentist's plaster made to proper consistence poured in and allowed to set. When this matrix is removed from the type, a piece of vulcanized rubber about ⅛ to 1 inch thick is cut to the size of the plaster and laid upon it. Both are then placed in a suitable screw-press, and heat is applied so as to thoroughly soften the rubber. The screw is then turned down hard, and left so for a time until the impression is well set. The rubber is then cemented to a block of wood, and the stamp is ready for use.—*Chem. and Drug.*

ARTIFICIALLY COLORED CIGARS. —It is alleged that the famous Madura cigars, remarkable for their fine, uniform brown color, which is indicative of an excellent quality of leaf, are colored by means of aniline dyes. New aniline is corrosive, and its effects can only be injurious to the smoker.

We think that this color, if it is at all employed for cigars, is applied in the country of production from which the choice cigars are exclusively obtained.—*Mon. prod. chim.*, 1884, *No.* 23, *p.* 355.

BOTTLE GLUE.—A good bottle glue, insoluble in water and particularly suitable for sealing bottles containing volatile liquids, such as chloroform, ether, alcohol, etc., may be prepared by soaking glue or gelatin in water, dissolving it in glycerin, then adding tannin (about 2 oz. for every pound of glue), and heating the mixture on a water bath until perfectly homogeneous, and as free from excess of water as possible. It may be colored if desired. When wanted for use, it is melted and applied to the mouth of the bottles.—*Trop. Agriculturist.*

SUBSTITUTE FOR WOMAN'S MILK.—The following is the formula suggested by Prof. Leeds of Stevens Institute, as the best substitute for woman's milk: 1 gill of cow's milk, fresh and unskimmed; 1 gill of water; 2 tablespoonfuls of rich cream; 200 grains of milk sugar; 1¼ grains of extractum pancreatis; 4 grains of sodium bicarbonate. "Put this in a nursing bottle, place the bottle in water made so warm that the whole hand cannot be held in it without pain longer than one minute. Keep the milk at this temperature for twenty minutes. The milk should be prepared just before using." —*Archives of Ped.*

BOROGLYCERIDE. —*H. F. S.* (*Philadelphia, Pa.*)—The following is a good practical formula :

| | |
|---|---|
| Glycerin, | 92 parts. |
| Boracic acid, pure | 62 parts. |

Mix and boil in a porcelain dish, or better a porcelain lined iron kettle. Stir continually with a glass rod until the mixture falls in "strings" from the end of the stirring rod when it is held above the kettle, or until the substance hardens quickly when a drop is placed on a cold porcelain surface. The preparation is then finished. It should be poured into cups or pots while still hot.—*Druggist Circular.*

TO REMOVE RUST FROM MARBLE.—An operation which depends upon the solubility of iron sulphide in a solution of potassium cyanide is thus effected: Clay is made into a thin paste with ammonium sulphide and the rust spot smeared with the mixture, care being taken that tne spot is only just covered. After a lapse of ten minutes this paste is washed off and replaced by one consisting of white bole mixed with a solution of potassium cyanide (1:4), which is in its turn washed off after a lapse of about 2½ hours. Should a reddish spot remain after washing off the first paste, a second layer may be applied for about five minutes.—*Pharmaceutische Centralhalle.*

CEMENT FOR GLASS, PORCELAIN, ETC.—Take some old soft cheese, and grind it in a mortar, washing it well at the same time with hot water. After the soluble matter is all washed away, a white mass of nearly pure caseine will remain. This should be squeezed in a cloth to express moisture, dried, reduced to powder, and preserved in a closely stopped bottle. When required for use, a small quanity should be ground up with a very little water, enough to make a thick, viscid paste, which must be used at once as it hardens quickly, and no heat should be used. Only such a quantity should be mixed as may be immediately needed, as, after it once hardens, it will not dissolve; and neither heat nor moisture has any effect upon it.—*Pop. Sci. News.*

PLASTIC CLAY FOR SUPPOSITORIES.—As a convenient material for suppositories in some cases Dr. Trippier recommends the use of plastic clay. The ordinary sculptors' modelling clay is used, the medicaments being dissolved in water, and then worked into the mass; in this way salts of iron and copper, alum, or even vegetable extracts may be incorporated by taking proper precaution. Dr. Trippier appears to contemplate supplying patients with the medicated clay so that they can break off a portion and mould it between the fingers as required. Although the mass may easily be maintained of a proper consistence in a vessel placed in a plate containing water and covered by a bell-glass, it is liable to harden if exposed in the open air; but this may be prevented by the use of glycerin, which is said to have the additional advantage of giving stability to a potassium iodide mixture. The formula given for such a mass is: Clay, 500 grms; water, 50 grms. potassium iodide, 30 grms. glycerin, 100 grms.—*Stearns' New Idea.*

